# Comparison of NREM sleep and intravenous sedation through local information processing and whole brain network to explore the mechanism of general anesthesia

**DOI:** 10.1371/journal.pone.0192358

**Published:** 2018-02-27

**Authors:** Yun Li, Shengpei Wang, Chuxiong Pan, Fushan Xue, Junfang Xian, Yaqi Huang, Xiaoyi Wang, Tianzuo Li, Huiguang He

**Affiliations:** 1 Department of Anesthesiology, Beijing Tongren Hospital, Capital Medical University, Beijing, China; 2 Research Center for Brain-inspired Intelligence, Institute of Automation, Chinese Academy of Sciences, Beijing, China; 3 The State Key Laboratory of Management and Control for Complex Systems, Institute of Automation, Chinese Academy of Sciences, Beijing, China; 4 Department of Anesthesiology, Plastic Surgery Hospital, Chinese Academy of Medical Sciences and Peking Union Medical College, Beijing, China; 5 Department of Radiology, Beijing Tongren Hospital, Beijing, China; 6 School of Biomedical Engineering, Capital Medical University, Beijing, China; 7 Sleep Medical Center, Department of Laryngology, Beijing Tongren Hospital, Beijing, China; 8 Beijing Shijitan Hospital, Capital Medical University, Beijing, China; Massachusetts General Hospital, UNITED STATES

## Abstract

**Background:**

The mechanism of general anesthesia (GA) has been explored for hundreds of years, but unclear. Previous studies indicated a possible correlation between NREM sleep and GA. The purpose of this study is to compare them by in vivo human brain function to probe the neuromechanism of consciousness, so as to find out a clue to GA mechanism.

**Methods:**

24 healthy participants were equally assigned to sleep or propofol sedation group by sleeping ability. EEG and Ramsay Sedation Scale were applied to determine sleep stage and sedation depth respectively. Resting-state functional magnetic resonance imaging (RS-fMRI) was acquired at each status. Regional homogeneity (ReHo) and seed-based whole brain functional connectivity maps (WB-FC maps) were compared.

**Results:**

During sleep, ReHo primarily weakened on frontal lobe (especially preoptic area), but strengthened on brainstem. While during sedation, ReHo changed in various brain areas, including cingulate, precuneus, thalamus and cerebellum. Cingulate, fusiform and insula were concomitance of sleep and sedation. Comparing to sleep, FCs between the cortex and subcortical centers (centralized in cerebellum) were significantly attenuated under sedation. As sedation deepening, cerebellum-based FC maps were diminished, while thalamus- and brainstem-based FC maps were increased.

**Conclusion:**

There’re huge distinctions in human brain function between sleep and GA. Sleep mainly rely on brainstem and frontal lobe function, while sedation is prone to affect widespread functional network. The most significant differences exist in the precuneus and cingulate, which may play important roles in mechanisms of inducing unconciousness by anesthetics.

**Trial registration:**

Institutional Review Board (IRB) ChiCTR-IOC-15007454.

## Introduction

Propofol is one of the most commonly used hypnotic agents for general anesthesia (GA). It can cause dose-related, controllable and reversible loss of consciousness (LOC), which is called sedation. Manifestations of sedation such as calm, drowsy, muscle relaxed and dreaming, are behaviorally similar to features of NREM sleep.[1] Therefore, efforts have been made to explore the correlations between these two statuses to explore the underline mechanism of general anesthesia.[2] If they are referred to the same functional brain areas, those brain areas could well be the control points of consciousness. On the other hand, the brain areas that propofol act different from sleep might be the specific targets that would contribute to the further research of GA mechanism.

Propofol can increase agonist efficacy at GABA receptors in the thalamic reticular nucleus which has been established to promote sleep.[3, 4] On the other side, it also alters central cholinergic neurotransmission to restrain cortical arousal.[5] Furthermore, the histaminergic tuberomammillary nucleus (TMN) and ventrolateral preoptic nucleus (VLPO) of posterior hypothalamus playing important roles in neural circuits of sleep/wake regulation recently have been shown involving in general anesthesia.[6, 7]

In addition, there seems to be remarkable similarities between propofol induced LOC and non-rapid-eye-movement (NREM) sleep in neuroimaging manifestation.[8, 9] It has been shown that propofol could dose-dependently reduce regional cerebral blood flow (rCBF) in the thalamus, midbrain, cuneus precuneus, posterior cingulate, and orbitofrontal cortex, which are less active during sleep than awake.[10–12] A recent EEG study on rats indicated that disruption of high γ frontal-parietal directed connectivity existed both in sleep- and anesthetic- induced unconsciousness.[13] There hasn't been such a study directly comparing of GA and sleep on human brain yet.

Several investigations have revealed that the integration of cortical and subcortical connection reversibly broken down and disruption of thalamocortical communication maybe the essential mechanism of anesthetic-induced LOC.[14] [15] There might be one or multiple brain areas acting as “key” to permitting or preventing subcortical-cortical processing.[16] Such “key” hasn’t been identified, and the connections of different brain areas under anesthesia are still not well understood.[17]

In this study, the resting-state fMRI was used to compare normal sleep and propofol induced sedation (PIS) on regional brain function and global functional networks. The aim was to find out the distinction and connection of the major stereotaxic sites and neural conductional pathway between sleep and PIS, so as to find out the brain areas which affect consciousness most and which are more relevant to GA, finally clarify whether GA and sleep are analogs.

## Materials and methods

The study was approved by the Ethics Committee of Beijing TongRen Hospital Ethics Committee No. TRECKY2003-007, Chairperson Prof. Ningli Wang and complied with the guidelines of the Declaration of Helsinki (1996). Before each section, a fully informed written consent was acquired. The study protocol had been registered in Chinese Clinical Trial Registry at November 25, 2015 (http://www.chictr.org.cn; Identifier: ChiCTR-IOC-15007454). Participants were recruited to the study and the data was collected from December 1, 2015 to November 30, 2016.

### Clinical protocol

#### Participants

Participants were enrolled by paid solicitation during December 1, 2015 to November 30, 2016. The include criteria were as follow: All subjects were medically screened by physical examination and mental evaluation (evaluated by Wechster Adult Intelligence Scale-Revised China (WAIS-RC)) before study, they were physically and psychiatric healthy; All the volunteers were fit and well (BMI<30, American Society of Anesthesiology gradeⅠ), right handedness, and had no history of food or drug allergy. The participants were excluded if there have any of following situations: taking medication or drugs acting on the central nervous system for therapeutic or drug addicts; Pontic, dental filling, metallic implant, claustrophobia, applying cosmetics and the females were not in menstrual period and menopause. In total 24 participants were successfully included. 12 participants who could easily fall asleep, 6 males and 6 females (Mean age 27, SD 7.3), were assigned to the sleep group (group S), and another 12 participants (Mean age 24, SD 3.8) with identical sex ratio were included in the PIS group (group P).

Before study, the details of the study were explained for each recruited participants. Furthermore, all participants were informed that they had the right to decline from participation. After informed consent was obtained, participants in the group P adhered to a standard pre-anesthesia protocol; they were told not to take solids for 8 hours and fluids for 4 hours before each experiment. After study, they were observed until fully recovered (Post-Anesthetic Discharge Scoring System (PADS) scoring > 9), then discharged with companions. We had access to information that could identify individual participants after data collection.

#### Physiological monitoring

The subjects were continuously monitored during the experiment. Heart rate, noninvasive blood pressure and oxygen saturation were measured continuously using a vital signs monitoring system (Invivo 3155MVS, Millennia Ltd, USA) and end tidal carbon dioxide concentration (P_ET_CO_2_) was detected by a multigas monitor (Drager Vamos 2, Germany).

#### Experimental protocol

For group P, an intravenous access was established (22G Introcan Safety-w, B. Braun Ltd). Throughout the experiments, oxygen was administered at 3 L/min via a face mask connected to MR anesthesia machine, and P_ET_CO_2_ was monitered via a nasal cannula. Two dedicated veteran anesthesiologists were responsible for the administration of propofol and monitoring of physiological parameters, respectively.

For each subject, a functional magnetic resonance imaging (fMRI) scanning was conducted at least 15 minutes after lying down with their eye-closed, relax and empty-headed as preanesthesia control state. A 1% propofol injection(Diprivan, AstraZeneca UK Limited) was administered using a target-controlled infusion pump (Graseby 3500 pump, Smiths Medical International Ltd., UK) programmed with Marsh model.[18] The target concentration of propofol was set at an initial value of 0.5 μg/ml. When effect site concentrations of propofol predicted from the Diprifusor model was arrived at the assuming ones and a 5-min pause was allowed for equilibration. Sedation states were evaluated by a specified surveyor using Ramsay scoring system.[19] Then, fMRI scanning was started. After that, the target concentration of propofol was increased 0.5 μg/ml gradually. After reaching a new stable effect site concentration, the fMRI scanning was again done until the defined clinical end points were achieved.

For each subject, the levels of sedation were evaluated by Ramsay Sedation Scale separately when the target effect-site concentrations were achieved and at the time after a 5-min equilibration period. A specialized person executed the procedure. When the Ramsay was 6, propofol infusion was stopped and the patient was observed until full recovery was achieved.

For group S, both drug and liquid were not infused. Scanning was conducted during daytime without sleep deprivation, and each participant lay on the examination couch comfortably with head fixation and ear clogged, and was told to relax and not to move. The fMRI scanning was performed at a sober quiet state and NREM sleep stage 2 (N2), respectively. The two states were determined by EEG.

#### EEG monitoring

Disposable carbon electrodes were applied on the scalp (C3, C4, P3, P4) according to the International 10–20 system (Jasper, 1958) for EEG and then connected to nonferrous fiber optic cables placed at the entrance to the bore of the magnet (MP150, BIOPAC Systems, Inc.). The fiber optic cables transmitted the signals out of the scanner room to a Neurolink Patient Monitor that reconstructed the analog EEG signals. By the low-pass filtering at 125 Hz, artifacts induced by MRI gradient magnetic field were removed based on template subtraction.[20] Subsequently, band-pass filtering was performed using a frequency range of 0.5–35 Hz. All procedures were performed using Acknowledge 4.2.0 for MP System software (BIOPAC Systems, Inc.).

#### Functional magnetic resonance imaging protocol

Participants were scanned in a 3 Tesla human Magnetic Resonance Imaging system (3.0T Signa HDx, GE medical system, LLC, USA). The fMRI data were acquired using the same MR system and echo planner imaging (EPI) sequence sensitive to blood oxygenation level-dependent(BOLD) contrast: repetition time(TR) = 2000ms, echo time(TE) = 35ms, flip angle(FA) = 90°, matrix = 64*64, resolution = 3.75×3.75mm, slices thickness = 5mm with interslice gap = 1 mm. Each brain volume comprised axial 28 slices, and each scanning session lasted for 400s. Sagittal T1-weighted MR image were acquired by a magnetization-prepared rapid gradient-echo sequence: TR = 8.876ms, TE = 3.516ms, FA = 13°, matrix = 256*256, FOA = 256*224, 196 continuous sagittal slices with 1mm thickness.

### Data analysis

#### Determination of sleep stages

EEG datasets were real-time observed by a sleep expert who had been working at Center for Sleep Sciences and Medicine of Beijing Tongren Hospital as an attending for two years. The sleep stages [wakefulness (W), stage 1, stage 2, slow wave sleep (SWS) or rapid eye movement (REM)] were judged for each 30 seconds interval. During the non-REM (NREM) sleep, EEG is characterized by lower frequencies (less than 8 Hz), computed over 2-min intervals. Stage N1 is defined as when θ wave (4~7 Hz) occupies more than 50% populations and vertex sharp wave appears. In the stage N2, θ wave accounts as majority for epochs with K complexes and sleep spindles go between. In the SWS, δ wave is >20% (0.5~2 Hz, >75μV). During the REM, α wave and saw tooth wave occasionally appear among θ waves.[21]

#### fMRI preprocessing

The first 10 volumes in the time series were discarded to avoid nonequilibrium effects in the MR signal. Then functional images were slice-time corrected, reoriented realigned, and unwarped to be corrected for susceptibility-by-movement interaction, resampled (1-mm isotropic), rigidly coregistered to anatomical template of the MNI space, and smoothed (Gussian kernel, 4mm full width at half maximum) using SPM (http://www.fil.ion.ucl.ac.uk/spm/) and DPARSF (http://www.restfmri.net/forum/). The movement parameters resulting from rigid body correction for head motion were removed by regression, as well as the global signal. Global signals from the image to rule out any confounding effect due to physiological (e.g. repiratory and cardiac) changes associated to propofol administration were also regressed out. Band-pass temporal filtering (0.01–0.08Hz) was used to remove magnetic field drifts the scanner and to minimize physiological noise of high-frequency components. Furthermore, the mean value or linear trend from the voxel time series were detrended.

#### ReHo analysis

Regional homogeneity (ReHo) is a measure of similarity or homogeneity of the series in a local neighborhood of voxels.[22] ReHo is the defined as the Kendall’s coefficient of concordance (KCC) of the time series of a given voxel with those of its nearest neighbors (26 in the current study). A lager ReHo value for a given voxel indicates higher regional coherence. It is defined formally as Kendall’s coefficient concordance:

$$\mathrm{ReHo}=\frac{\sum {\left(R_{i}{\bar{R}}_{i}\right)}^{2}}{K^{2}\left(n^{3}-n\right)/12}=\frac{\sum {\left(R_{i}\right)}^{2}-n{\left({\bar{R}}_{i}\right)}^{2}}{K^{2}\left(n^{3}-n\right)/12}$$ (1)

Where, $R_{i}=\sum_{j=1}^{k}r_{ij}$ is the sum rank of the *i*th time point and *r*_*ij*_ is the rank of the *i*th time point of the *j*th voxel: where ${\bar{R}}_{i}$ is the mean of the *R*_*i*_; over all time point *i*; *N* is the length of time series; and *k* is the number of voxels within the “neighborhood” of each index voxel (k = 27 in the present study). Reho ranges from 0 to 1, which the higher values indicating greater similarity of time series in the local neighborhood.

Maps of Reho were estimated for each voxel and standardized within each subject to generate Z-score maps, which could be appropriately averaged and compared across participants. The z-score of a voxel was calculated simply by subtracting the whole map mean and dividing by the whole map standard deviation.

#### Seed-based connectivity analysis

Brain stem, thalamus and cerebellum were three most important subcortical centers, all neural signals must go through from one of them at least. So these three specific regions of interest were selected as seed points for further interrogate. The masks of cerebellum and thalamus were extracted based on AAL template, while the mask of brainstem was extracted based on HarvardOxford_Atlas in MNI standard space. These masks were then registered to each subject’s functional MNI space. The individual subjects’ mean BOLD signal time series of all voxels from each ROI were extracted from each of the functional scans respectively. Subsequently, the Pearson correlation coefficient (i.e. functional connectivity (FC)) between the mean time series of ROI and that of each voxels of the whole brain were calculated to obtain the seed-based FC map. (S1 Raw data)

#### Statistical analysis

Qualitative estimation of Reho and seed-based FC map changes were performed by statistical comparisons among different states. In terms of the purpose and design of experiment, the different statistical methods were used. (1) A paired *t* test was performed to identify significant differences of areas in the group of sleep; (2) A repeated-measures ANOVA was performed to identify significant differences of areas among wake, m-PIS, and d-PIS. (3) Two sample *t* test was carried out to compare the state of sleep with m-PIS or d-PIS.

## Results

### EEG and physiologic variables

Age, gender and body mass of two groups were regressed as covariates. Despite the sleep condition in MRI room was uncomfortable because of persistent noise, fixed head and body position, hard bed, and daytime hours without sleep deprivation, all 12 subjects in the group S were able to fall asleep within two hours by an inquisition whether slept or not after scanning to double check. One participant was excluded from further analysis due to apparent motion artifact (translated >2 mm or rotated >2°in the motion correction during the image registration procedure). All of the remaining 11 EEG data sets in the group S presented continuous segments for at least 10 min of wakefulness and 10 min of N2 sleep per subject. Under the two status, resting state fMRI scanning was accomplished.

**Fig 1:**
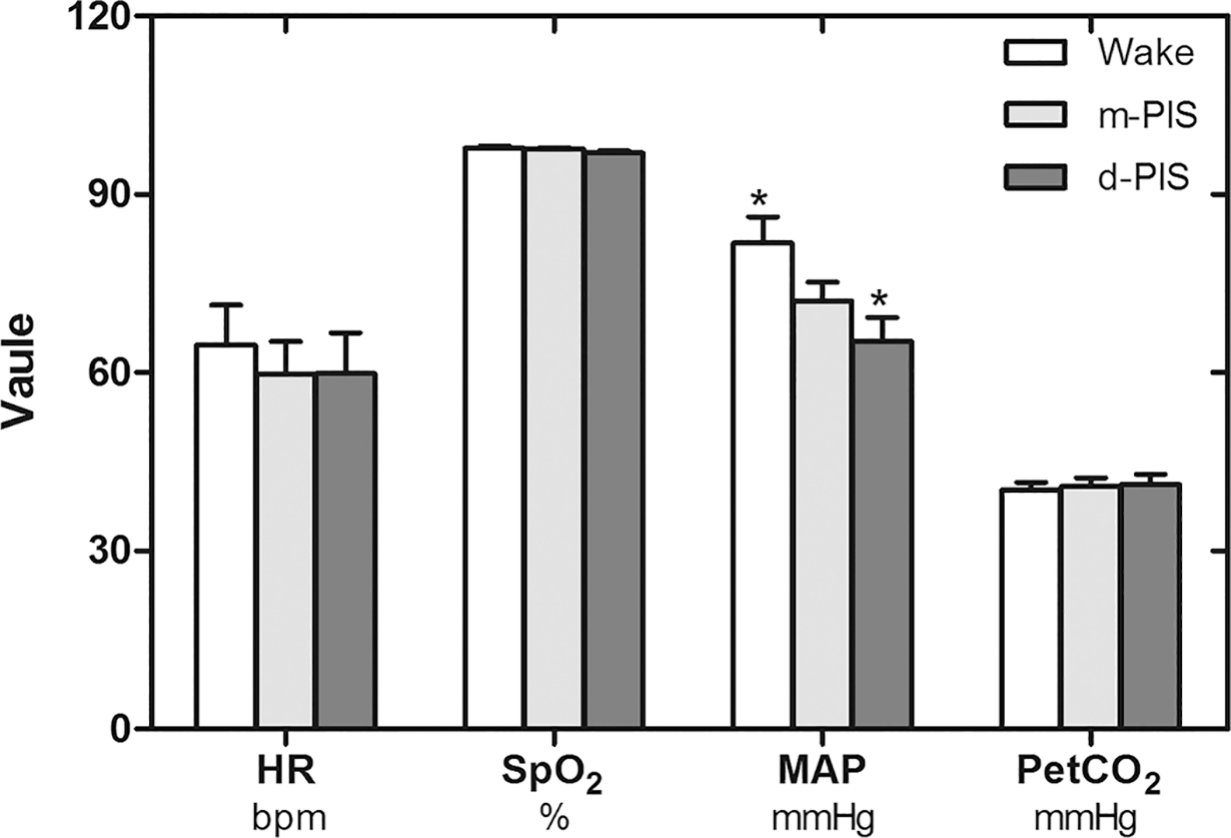
Comparisons of physiological variables in wake, m-PIS and d-PIS states (N = 12). There were no significant differences in HR, SpO2, PetCO2 between states, but significant differences in MAP between wake and d-PIS states (repeated-measures ANOVA, * P<0.01, compared with wake state). HR, heart rate; SpO2, oxyhemoglobin saturation; MAP, mean artery pressure; PetCO2, end-tidal carbon dioxide.

Several pieces of MRI data in the group P were ruled out during each step by increasing level of sedation because of violent body movement, but the previous data before moving was reserved. Most of physiological variables in the group P were stable throughout the experiment except blood pressure at two levels of propofol sedation. Significant MAP differences were observed among waking, mild sedation and deep sedation stages (Fig 1).

### ReHo analysis

**Fig 2:**
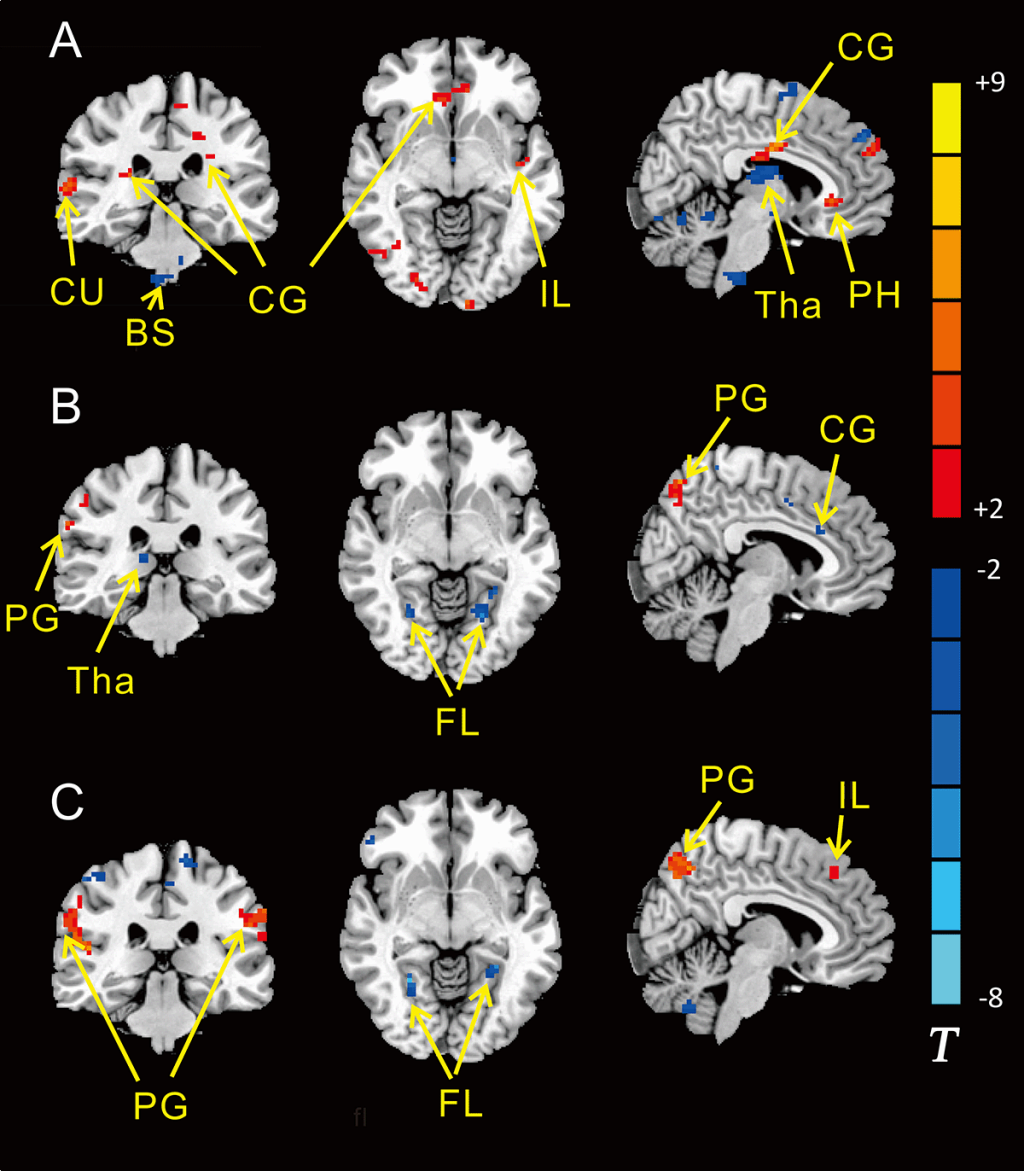
ReHo of different brain regions in wake, sleep, m-PIS and d-PIS states. A showing significantly differences between sleep and wake states (N = 11); B showing significantly differences between m-PIS and wake states (N = 12); C showing significantly differences between d-PIS and wake states (N = 12) (Two sample t-test, P<0.01, cluster size>19). The blue regions represent significantly increased ReHo values and the red regions represent significantly decreased ReHo values in the sleep, m-PIS and d-PIS states compared to the wake state. CU, cuneus; BS, brainstem; CG, cingulate gyrus; IL, insula lobe; Tha, thalamus; PH, parahippocampal gyrus, PG, precuneus gyrus; FL fusiform lobe. Color indicates t values. The larger |t| indicates more variation.

As for local metrics, results for nodal strength are shown in Fig 2, Tables 1–3. Compared to default mode (wakefulness), the initial stage of NREM sleep had a significant weakening on cortex of frontal lobe, especially preoptic area, semi-lunar and cingulate lobule, but an enhancement on limbic lobe, parahippocampal gyrus, thalamus, cerebellum and pons.

**Table 1:** Comparisons of ReHo between waking and sleep states.

Peak MNI coordinate region	Voxels	Peak MNI coordinate			Peak intensity
		x	y	z	
***Waking < Sleep*, *P<0*.*01*,*uncorrected*, *cluster size >20***					
Superior Frontal Gyrus	24	6	51	36	-5.1489
	27	-15	-15	72	-3.1309
Cerebellum Anterior Lobe	26	12	-54	-18	-4.5109
Cerebellum Posterior Lobe	42	-36	-75	-30	-4.7786
	34	30	-45	-39	-4.5508
Inferior Parietal Lobule	19	-63	-39	36	-3.4281
Limbic Lobe, Fusiform Lobe, ParaHippocampal Lobe	46	-36	-12	-33	-4.671
	63	27	3	-24	-4.3986
Medial Frontal Gyrus	50	12	-6	60	-4.9063
	25	18	48	15	-4.7683
Middle Frontal Gyrus	29	57	24	33	-3.2835
Pons	20	-6	-24	-39	-4.6617
Posterior Cingulate Gyrus	27	-9	-60	3	-3.3063
Superior Temporal Gyrus	21	-48	-57	21	-3.7914
Thalamus	96	6	-21	12	-4.5156
Vermis	37	3	-66	-9	-4.09
***Waking > Sleep*, *P<0*.*01*,*uncorrected*, *cluster size >20***					
Cingulate Gyrus	68	6	30	-3	4.3905
	23	6	-6	33	5.4677
Cerebellum Posterior Lobe	20	-18	-69	-39	5.3468
Inferior Frontal Lobe	37	42	27	-21	4.2992
Inferior Semi-Lunar Lobe	20	9	-72	-51	3.8274
	70	-24	-78	-54	7.2331
Limbic Lobe	26	21	6	-42	4.3027
Medial Frontal Gyrus	39	-9	33	30	4.7311
Middle Frontal Gyrus	131	27	27	36	4.6862
	75	-30	39	39	5.3086
	30	-24	12	51	5.369
	26	33	48	9	4.6334
Occipital Lobe	74	21	-78	3	3.65
	22	-9	-102	-6	4.5178
Postcentral Gyrus	30	-54	-15	51	5.6272
Superior Temporal Lobe	21	63	-30	9	4.5002
	28	-48	-9	-3	3.7674

**Table 2:** Comparisons of ReHo from different brain areas between waking and m-PIS states.

Peak MNI coordinate region	Voxels	Peak MNI coordinate			Peak intensity
		x	y	z	
***Waking < m-PIS*, *P<0*.*01*,*uncorrected*, *cluster size >19***					
Cingulate Gyrus	29	3	21	27	-4.9798
	26	-6	9	42	-4.2235
Cerebellum Posterior Lobe	43	-12	-63	-27	-4.6556
Fusiform Lobe	37	33	-60	-12	-5.8612
Lingual Lobe	21	-24	-66	-3	-5.5727
Postcentral Gyrus	70	30	-45	54	-6.4378
Precentral Gyrus	19	36	-18	69	-3.9695
Thalamus	21	18	-21	12	-5.1599
***Waking > m-PIS*, *P<0*.*01*,*uncorrected*, *cluster size >19***					
Inferior Frontal Gyrus	23	51	27	15	4.0409
Inferior Parietal Lobe	25	-63	-36	36	3.4862
Insula	25	39	-9	9	6.685
Middle Frontal Gyrus	32	-33	48	0	4.3732
	24	27	51	24	6.8078
	24	-42	9	39	6.4358
Middle Temporal Gyrus	28	-36	-57	18	8.4362
Precentral Gyrus	70	51	-6	12	6.4093
Precuneus Lobe	27	6	-69	57	5.515
Superior Frontal Gyrus	22	30	42	45	4.7826
	21	-15	48	39	4.4869
Superior Temporal Gyrus	41	-60	3	6	6.2505
SupraMarginal Lobe	33	60	-21	18	6.4674
	60	54	-18	27	6.2626

**Table 3:** Comparisons of ReHo from different brain areas between waking and d-PIS states.

Peak MNI coordinate region	Voxels	Peak MNI coordinate			*Peak intensity*
		x	y	z	
***Waking < d-PIS*, *P<0*.*01*,*uncorrected*, *cluster size >19***					
Cerebellum Posterior Lobe	36	9	-60	-39	-5.3
Fusiform Lobe	46	27	-54	-6	-6.9458
Inferior Frontal Gyrus	20	57	39	3	-5.4197
Lingual Lobe	43	-30	-45	-3	-8.6288
Paracentral Lobe	54	-9	-42	60	-8.0003
	19	9	-33	66	-6.0788
	25	-9	-27	66	-5.843
	24	51	-27	60	-5.6282
***Waking < d-PIS*, *P<0*.*01*,*uncorrected*, *cluster size >19***					
Cingulate Gyrus	20	0	-6	36	4.8207
	23	-6	-60	15	3.5041
Inferior Frontal Gyrus	34	33	24	9	6.0504
Inferior Temporal Gyrus	22	-54	-51	-18	3.4623
Insula	57	-36	15	6	4.9755
Middle Frontal Gyrus	101	27	42	24	5.5427
	39	-36	12	45	6.7931
	34	51	12	48	4.2459
Middle Temporal Gyrus	34	-39	-57	15	7.3076
Precuneus Lobe	105	57	-3	18	5.7171
	59	3	-69	54	6.326
Superior Frontal Lobe	39	-6	30	48	5.1333
SupraMarginal Lobe	172	60	-18	12	9.5363
	164	-63	-24	18	6.631

Under mild propofol induced sedation (m-PIS), the nodal strength on cingulate and postcentral gyrus, fusiform, lingual lobe, thalamus and cerebellum was increased, but the nodal strength on frontal lobe, supramarginal gyrus, precuneus and insula was attenuated in comparing with wake states as a self- control study. Under deep propofol induced sedation (d-PIS), the nodal strength across diverse locus of subcortical center close to midline regions was significantly inhibited, but the nodal strength on supramarginal gyrus, cingulate, precuneus, insula and some portion of frontal cortex was significantly increased in comparing with their own wake states.

**Fig 3:**
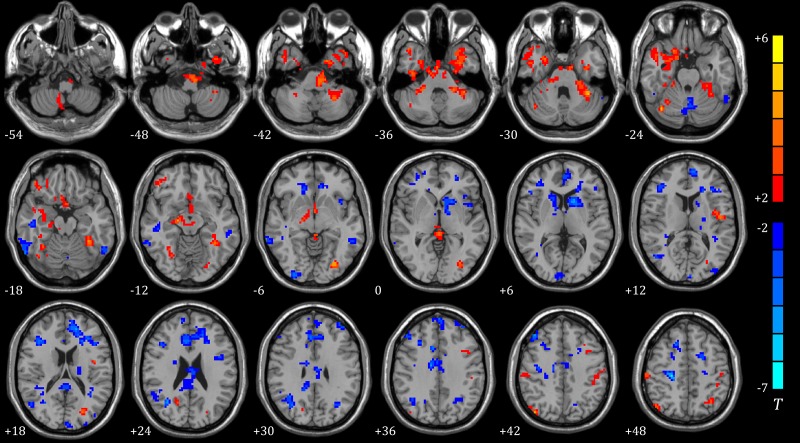
ReHo of different brain regions between sleep (N = 11) and m-PIS (N = 12) (two sample t-test, *P*<0.01, cluster size>19). The blue regions represent significantly increased ReHo values and red regions represented significantly decreased ReHo values in m-PIS state compared to sleep state. Color indicates t values.

**Fig 4:**
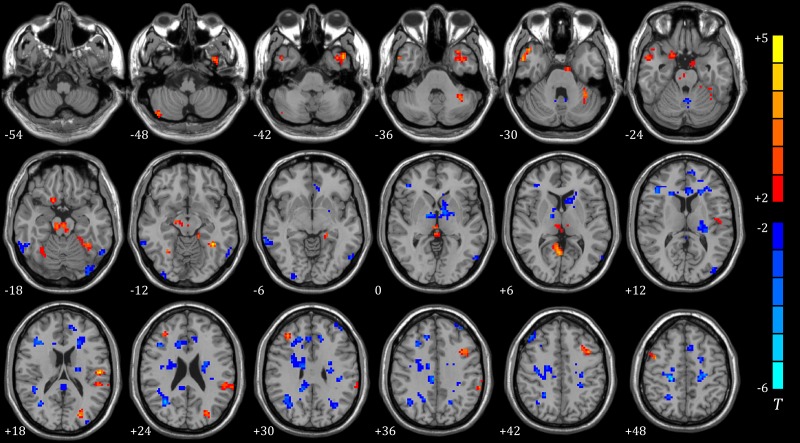
ReHo of different brain regions between sleep (N = 11) and d-PIS (N = 12) (two sample t-test, *P*<0.01, cluster size>19). Blue regions represent significantly increased ReHo values and red regions represented significantly decreased ReHo values in d-PIS state compared to sleep state. Color indicates t values.

Comparison with N2 sleep and PIS, brain areas with significant variance spread all over the brain, predominant centralized in subcortical area, cingulate and fusiform gyrus. Disparities between two sedative conditions and N2 sleep were roughly alike. (Shown in Figs 3 and 4, Tables 4 and 5)

**Table 4:** Comparisons of ReHo from different brain areas between sleep and m-PIS states.

Peak MNI coordinate region	Voxels	Peak MNI coordinate			Peak intensity
		x	y	z	
***Sleep < m-PIS*, *P<0*.*01*,*uncorrected*, *cluster size >19***					
Cingulate Gyrus	25	-9	33	-3	-3.7493
	306	12	-9	36	-5.1862
Cerebellum Posterior Lobe	55	-3	-66	-24	-3.6758
Cuneus	25	6	-99	6	-3.1801
	21	-3	-99	15	-3.771
Inferior Frontal Gyrus	24	-45	3	24	-3.3602
	136	42	24	21	-4.4658
Inferior Occipital Gyrus	31	30	-96	-6	-4.5867
Inferior Temporal Gyrus	40	-60	-57	-18	-3.5271
	103	60	-63	-18	-4.2638
Insula	29	-39	12	-6	-3.2877
Middle Frontal Gyrus	51	42	36	42	-4.9979
	26	30	24	36	-3.5156
Middle Occipital Gyrus	24	-39	-90	12	-2.8523
	39	-33	-66	33	-3.7758
Middle Temporal Gyrus	44	-54	-45	-6	-3.4512
	160	33	-60	24	-4.983
Superior Frontal Gyrus	62	0	60	36	-4.0506
	26	18	21	48	-3.8182
	55	-9	9	69	-4.9843
	35	-36	51	30	-3.5137
Superior Temporal Gyrus	20	-36	-51	18	-3.3082
***Sleep < m-PIS*, *P<0*.*01*,*uncorrected*, *cluster size >19***					
Cerebellum Anterior Lobe Pons	24	12	-51	-51	3.4882
Cerebellum Posterior Lobe	32	39	-69	-24	5.0092
Fusiform Lobe Middle Temporal Gyrus	228	-30	-3	-36	3.583
	45	33	-54	-15	3.669
	950	-36	-48	-15	5.6819
Inferior Frontal Gyrus	68	45	39	-15	4.7819
Inferior Occipital Gyrus	47	-30	-81	-3	5.1366
Middle Frontal Gyrus	23	-36	12	39	3.0037
Middle Temporal Gyrus	183	45	18	-33	3.708
Postcentral Gyrus	25	60	-24	48	3.415
	38	-42	-36	45	2.8601
Precuneus Gyrus	22	21	-60	21	3.3074
	33	-6	-66	57	3.9358
Superior Parietal Lobe	210	21	-54	72	5.4373
	177	-24	-48	72	6.1286
	33	-33	-63	51	3.827

**Table 5:** Comparisons of ReHo from different brain areas between sleep and d-PIS states.

Peak MNI coordinate region	Voxels	Peak MNI coordinate			Peak intensity
		x	y	z	
***Sleep < d-PIS*, *P<0*.*01*,*uncorrected*, *cluster size >19***					
Anterior Cingulate Lobe	24	-12	33	30	-3.2333
Cerebellum Anterior Lobe	28	-3	-57	-27	-3.4098
Inferior Frontal Gyrus	96	42	21	21	-4.4013
Inferior Occipital Gyrus	32	-33	-93	-15	-3.3367
	25	33	-93	-9	-2.9177
Inferior Parietal Lobe	21	-36	-30	27	-3.4359
Inferior Temporal Gyrus	71	54	-48	-21	-3.7146
Medial Frontal Gyrus	126	15	33	36	-3.998
	36	-18	45	15	-3.3014
	19	-6	18	48	-2.8711
	26	45	36	42	-4.0983
Middle Occipital Gyrus	52	-57	-66	-9	-3.1681
	28	-33	-66	30	-3.2116
Parietal Lobe	129	33	-60	24	-5.0711
Precentral Gyrus Cingulate Gyrus	422	24	-24	51	-6.3056
	40	24	-51	42	-4.1092
Superior Frontal Gyrus	20	-36	48	36	-3.4381
Superior Occipital Gyrus	21	-33	-93	21	-3.034
***Sleep < d-PIS*, *P<0*.*01*,*uncorrected*, *cluster size >19***					
Cerebellum Posterior Lobe	21	45	-75	-45	3.7971
Fusiform Lobe	94	-36	-48	-15	4.7166
Inferior Frontal Gyrus	49	-39	12	39	4.8179
Inferior Parietal Gyrus	65	-63	-39	36	3.0177
Inferior Temporal Gyrus	89	-45	9	-42	4.0747
Middle Frontal Gyrus	29	36	36	30	3.7045
	19	45	3	48	3.8294
	47	27	-6	63	4.3
Middle Occipital Gyrus	41	-24	-81	18	3.6993
Postcentral Gyrus	31	-54	-18	18	4.4723
Posterior Cingulate Gyrus	44	3	-60	6	4.1149
Right Brainstem	59	9	-24	-18	3.3498
Superior Parietal Gyrus	157	-33	-51	66	4.5281
	80	21	-51	72	4.2088
Superior Temporal Gyrus	77	48	18	-30	3.8089

### Seed-based connectivity analysis

At the global cerebral level, the average connectivity matrices and the frequency distribution of correlations based on 3 ROIs were different among groups. To some extent, changes on FCs were bilateral symmetry.

**Fig 5:**
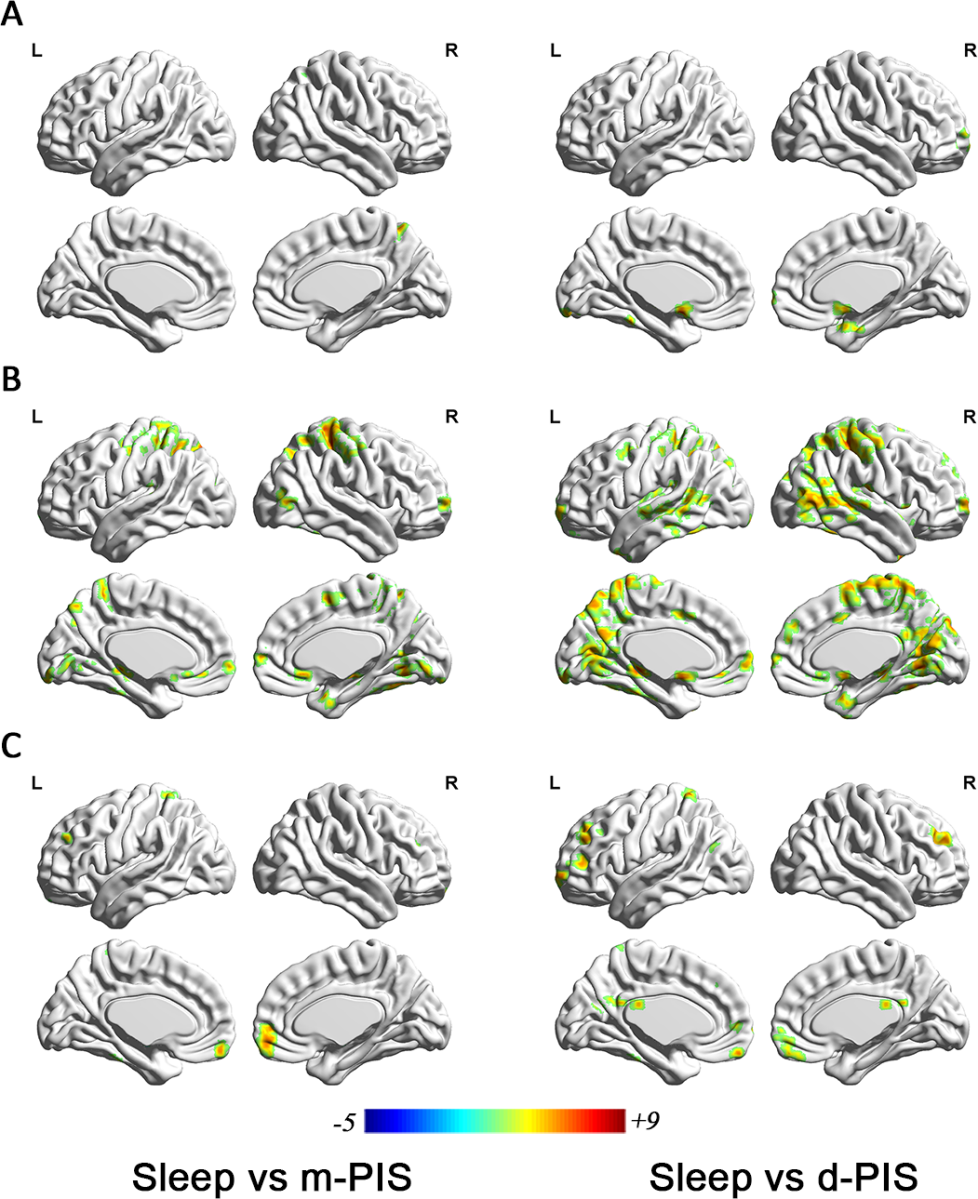
Differences in the FC maps based on three ROIs between sleep (N = 11) and m/d-PIS (N = 12) (*P*<0.01, cluster size>19). A showing significantly differences in the FC map based on brainstem between sleep and m-PIS (left) /d-PIS (right); B showing significantly differences in the FC map based on cerebellum between sleep and m-PIS (left) /d-PIS (right); C showing significantly differences in the FC map based on thalamus between sleep and m-PIS (left) /d-PIS (right). Blue regions represent significantly increased FC values and red regions represent significantly decreased FC values in m/d-PIS state compared to sleep state. Color indicates t values.

Correlations between each ROI and the rest of global brain domain were unequally affected by N2 sleep and PIS (p<0.01, uncorrected). There was a significant difference between N2 sleep and m-PIS in FCmaps. FCs from precuneus, cingulate, insula, putamen, fusiform, parahippocampal and four main areas of the cortex to subcortical centers were significantly weakened in m-PIS compared to N2 sleep. In addition, difference in FCmaps between mild and deep sedation was not that notable. As sedation deepening, the cortex’s ends of FC alternation were most similar to mild sedation, but the subcortical ends had an upward trend, shifting from cerebellum to thalamus. Among those above-mentioned areas, the precuneus and cingulate cortex were the most prominent parts in the FC difference. (Shown in Fig 5, Tables 6 and 7)

**Table 6:** Comparisons of FC maps from different brain areas between sleep and m-PIS states.

Peak MNI coordinate region	Voxels	Peak MNI coordinate			Peak intensity
		x	y	z	
***ROI of Brainstem*, *P<0*.*01*, *cluster size>20***					
Precuneus	40	15	-57	57	5.1179
***ROI of Cerebellum*, *P<0*.*01*, *cluster size>20***					
Anterior Cingulate Gyrus	53	3	24	-9	4.8617
Posterior Cingulate	210	3	-75	-3	5.0115
Frontal Lobe	L	-12	18	-12	4.2876
	35	-27	-12	51	4.6664
	36	9	3	54	4.9479
	21	-3	66	0	3.9358
	62	9	63	6	4.7684
Fusiform Lobe	78	33	-57	-15	5.6931
Limbic Lobe	43	-15	-33	-6	6.1431
Occipital Lobe	51	-12	-96	-18	5.3056
	20	51	-75	3	5.0339
	63	-27	-75	12	4.6172
ParaHippocampal	20	27	6	-27	5.2639
Parietal Lobe Precuneus Lobe	416	-9	-75	54	5.2949
Pons	40	-18	-36	-33	4.2934
Postcentral Gyrus	443	39	-33	60	8.6185
Precuneus Lobe	270	21	-69	42	5.777
	27	-15	-69	36	4.2282
Putamen	23	36	0	0	4.5403
Superior Temporal Lobe	21	-51	-33	18	4.0261
***ROI of Thalamus*, *P<0*.*01*, *cluster size>20***					
Cerebellum Anterior Lobe	26	-18	-36	-36	5.7896
Middle Frontal Gyrus	22	-27	45	33	3.7969
	104	-6	57	-18	5.6493
	25	24	36	24	5.4405
Superior Parietal Lobe	22	-30	-48	69	4.0787

**Table 7:** Comparisons of FC maps from different brain areas between sleep and d-PIS states.

Peak MNI coordinate region	voxels	Peak MNI coordinate			Peak intensity
		x	y	z	
***ROI of Brainstem*, *P<0*.*01*, *cluster size>20***					
Anterior Cingulate Lobe	78	-6	6	-12	7.212
Cerebellum Anterior Lobe	25	-21	-42	-30	4.6012
	70	15	-54	-18	5.1825
	20	21	-66	-60	4.7367
	34	48	-69	-27	4.1657
Frontal Lobe	20	30	66	6	3.5082
	27	15	72	-3	4.1188
Occipital Lobe	31	-12	-90	-12	3.5774
ParaHippocampal	71	27	-9	-27	5.3691
***ROI of Cerebellum*, *P<0*.*01*, *cluster size>20***					
Pons	22	9	-18	-30	4.6647
Cingulate Gyrus	34	-9	6	42	3.6423
Frontal Lobe Putamen	170	-3	6	-12	5.5491
	184	12	21	-12	7.7024
	31	48	15	0	4.8335
	25	-42	9	48	3.7817
	34	9	48	39	5.0581
	149	21	66	6	5.1436
	69	36	-54	-21	5.6191
Insula	44	-30	-18	6	4.837
Occipital Lobe Lingual Gyrus	105	-12	-99	-18	4.5319
Parietal Lobe Occipital Lobe	3096	6	-39	69	6.3978
Precuneus Lobe	33	-33	-66	39	3.6363
Temporal Lobe Fusiform Gyrus	280	-42	-36	-21	5.0585
	60	30	6	-39	7.0443
	171	-39	-57	9	5.6492
Thalamus	96	12	-24	3	5.5672
	112	-12	-33	-6	6.8597
***ROI of Thalamus*, *P<0*.*01*, *cluster size>20***					
Cerebellum Posterior Lobe Cerebellum Anterior Lobe	47	39	-57	-57	5.9658
	30	9	-57	-51	4.6956
	71	-21	-36	-36	4.6837
	22	-33	-57	-33	4.5175
	32	-12	-45	-24	4.0497
Medial Frontal Gyrus	43	-3	57	-21	5.6541
	53	-21	63	-3	4.9359
Caudate	48	18	3	9	4.9166
Precuneus Lobe	56	-9	-66	21	4.3163
Middle Occipital Gyrus	32	-33	-63	27	4.3592
Posterior Cingulate	20	-3	-30	24	4.1918
Middle Frontal Gyrus	136	24	36	24	5.3442
	276	-21	42	33	6.0155
Superior Parietal Gyrus	32	-27	-48	69	6.2188

## Discussion

General anesthesia manifests like sleep. Anesthetists often tell their patients “just having a sleep” to explain the GA process. From an interpretivist perspective, the alterations of consciousness within GA and sleep are somewhat alike. Moreover, they have some similar molecular mechanisms. On the other side, different experiences and growth environments form different brain activities when participants awake. Even tiny turbulence affects fMRI results tremendously. By contrast, sleep is a relative stable status with less thinking activity disturbance comparing with the wake state. So we compared the spatio-temporal changes PIS with sleep on the clustering of instantaneous PCC-related spatial maps. Our study replenished literature of researches on neuromechanism of sleep and GA under functional neuroimaging especially in the global functional domain. The whole brain was considered as an integrity to understand what functional alternations and FC potencies were occurring from subcortical centers to cortex regions along with sleep and sedation happening.

For local brain function, NREM sleep mainly associated with frontal lobe (mostly preoptic area), cerebellum, thalamus, limbic/parahippocampal, cingulate, insula, cuneus and fusiform gyrus. Particularly noteworthy was that the brainstem function significantly strengthened. However, the propofol induced LOC influenced a large amount of brain areas, especially, cerebellum, thalamus, cingulate, fusiform, insula and precuneus gyrus. Among them, the cingulate, insula and fusiform gyrus were the same function changed regions during sleep and PIS what previous studies didn’t mention, excepting for the superior frontal lobe. However, local metrics alterations manifested much more differences than similarities between N2 sleep and mild sedation, indicated them to be completely separate physiological statuses on neurological function.

Seed based FCmaps showed that projections from the subcortical centers—cerebellum and thalamus to frontal lobe were significantly strengthened during sleep, but FC from brainstem to superior frontal lobe was weakened. Thus, we speculate that frontal lobe, especially the upper one, would be the most important brain area for regulating sleep. Furthermore, brainstem might be included in awakening circuit, whereas cerebellum and thalamus might be involved in sleep circuit. Whereas propofol primarily interfered whole brain FC network especially the subcortical-cortical connections, what was in accordance with previous studies.[14, 15] Another evidence to support our hypothesis was that the FC alterations were broadly consistent during mild and deep PIS in this study. The wild differences between sleep and PIS on FCmaps indicated that they shared little similarity. Precuneus and cingulate Gyrus based FCs were the most prominent distinctions between sleep and PIS, which might be involved in the key neural pathway of propofol sedation.

The precuneus projects ascendingly to somatosensory, cognitive and visual cortex, and participates in episodic memory, visuospatial processing, and proprioceptive sensations.[23] It connects to the subcortical nuclei including the ventral lateral nucleus, the intralaminar nuclei, and the lateral pulvinar, putamen, the nucleus reticularis tegmenti pontis, and the basis pontis, which have been proved to be involved in sleep or anesthesia by animal experiments.[24] Cerebral glucose metabolism in precuneus is at the highest level under wakefulness, but is mostly reduced during anesthesia. In addition, the precuneus is one of most deactivated brain areas during slow-wave sleep and rapid eye movement sleep.[25] Traditionally, the precuneus was considered as a homogeneous structure with the adjacent posterior cingulate, and those two adjacent brain areas were thought to be “hub” for conscious information processing.[24] Anterior cingulate cortex (ACC) has been described as an junction in neural network of maintaining consciousness,[25] which was also verified in rats.[26, 27] In a PET study, when the plasma concentration of propofol increases, rCBF in the precuneus and posterior cingulate decreased.[10] Local utilization of glucose was most prominently reduced in cingulate cortex among 35 regions of the rat brain under propofol anesthesia, achieving 76%.[28] Furthermore, propofol significantly reduces the FOS-LI positive cells in the posterior cingulate and retrosplenial cortices (PC/RS).[29] Unlike NREM, propofol caused a persisted increasing in gamma (25–40 Hz) power under high-density electroencephalography (hd-EEG), which was originated from the anterior and posterior cingulate cortices.[1] Sevoflurane could also reduce the posterior cingulated cortex and increase the posterior insula in the connectivity during administration.[30] Our results furtherly verified the cingulate cortex as a nerve center for maintaining consciousness during sleep and PIS, because its activities and FCs were significantly inhibited. In addition, the precuneus may be more associated with propofol sedation.

There are some limitations of present study. The first one is the criterion of evaluating the levels of sedation. The endpoints of each sedation status are broad and not consistent, so it’s unable to group precisely. However, physiological data to determine the level of sedation does not exist.[31] Further, the levels and qualities of sedation depend on the effect-site concentration which is based on a 3-compartment pharmacokinetic model.[32] At a lower dose, individuals could be evoked by sonic or tactile stimulus just as hypnoid, at a higher dose, however, subjects might be unresponsive to a noxious stimulus.[2] Those statuses can be classified by the Ramsay scoring system, which is sensitive, reliable and valid in measuring levels of sedation.[19] As for sedation scales, EEG correlates well with the Ramsay Scale.[33] Since the Ramsay sedation scale could merely provide a roughly estimating the depth of sedation, drug-effect grouping in this study didn’t represent exactly brain functional changes based on sedation depth.

Second, each scanning was conducted at daytime, so most of candidates could only reach a N2 sleep, or just concerned as a nap. Such a condition was unstable and festless sleep. Furthermore, there might be larger distinguish between deep sleep and PIS in the brain functional changes. The regret may be compensated by further studies. To define subjects falling asleep or not, only 4-lead EEG was applied because of lacking better measurement. It is well known that EEG is easily interfered. When MRI scanning was starting, the EEG waveform cannot be recognized. To address this issue, the EEG was assessed before and after MRI scanning immediately to ensure that the subjects had been fallen asleep. In addition, an inquiry about sleep or dream was accomplished after each experiment.

Third, one may argue that drug-induced changes in flow-metabolism coupling would have interfered with the neural changes shown in this study. However, blood pressures during propofol mild sedation did not significantly changed compared with their baselines. Moreover, previous studies have shown that propofol has no direct effect on cerebrovascular imaging and can preserve flow-metabolism coupling.[34] Thus, the results in BOLD connectivity observed in this study should reflect changes in neural activities.

Sometimes, unconsciousness induced by anesthetics can be reversed by voice or tap just like a real sleep, but the intrinsic neurophysiological mechanisms of those two statuses are far apart. Neither is a simple nor a describable process. Sleep mainly associates with brainstem and frontal lobe, while PIS can cause whole brain function changing. The superior frontal lobe shares some common both in local neural function and FC alternations under those two statuses. That maybe a breakthrough to discover the mystery of maintaining consciousness. While the differences between sleep and sedation especially in the precuneus and cingulate may provide evidences for us to penetrate deeply grope for the mechanism of GA in the future.

## Supporting information

S1 Raw Data Raw data of based-voxel functional connectivity in terms of ROIs. The zip file can be unzipped to a set of MATALB *.mat files, which contain based-voxel functional connectivity data and relevant information. The whole data set can be divided into two groups, i.e., sleep group and anesthesia group. The sleep group data includes two states (sleeping and waking), while the anesthesia group data includes three states (waking, mild-PIS and deep-PIS).
(ZIP)

## References

[1] Murphy M, Bruno MA, Riedner BA, Boveroux P, Noirhomme Q, Landsness EC, et al Propofol anesthesia and sleep: a high-density EEG study. Sleep. 2011;34(3):283–91a. Epub 2011/03/02. ; PubMed Central PMCID: PMCPmc3041704. 21358845 10.1093/sleep/34.3.283 PMC3041704

[2] Franks NP. General anaesthesia: from molecular targets to neuronal pathways of sleep and arousal. Nat Rev Neurosci. 2008;9(5):370–86. Epub 2008/04/22. doi: 10.1038/nrn2372 . 18425091 10.1038/nrn2372

[3] Solt K, Forman SA. Correlating the clinical actions and molecular mechanisms of general anesthetics. Curr Opin Anaesthesiol. 2007;20(4):300–6. Epub 2007/07/11. doi: 10.1097/ACO.0b013e32816678a5 . 17620835 10.1097/ACO.0b013e32816678a5

[4] Jones EG. Thalamic circuitry and thalamocortical synchrony. Philos Trans R Soc Lond B Biol Sci. 2002;357(1428):1659–73. Epub 2003/03/11. doi: 10.1098/rstb.2002.1168 ; PubMed Central PMCID: PMCPmc1693077. 12626002 10.1098/rstb.2002.1168 PMC1693077

[5] Meuret P, Backman SB, Bonhomme V, Plourde G, Fiset P. Physostigmine reverses propofol-induced unconsciousness and attenuation of the auditory steady state response and bispectral index in human volunteers. Anesthesiology. 2000;93(3):708–17. Epub 2000/09/02. . 10969304 10.1097/00000542-200009000-00020

[6] Luo T, Leung LS. Involvement of tuberomamillary histaminergic neurons in isoflurane anesthesia. Anesthesiology. 2011;115(1):36–43. Epub 2011/05/13. doi: 10.1097/ALN.0b013e3182207655 . 21562401 10.1097/ALN.0b013e3182207655

[7] Gaus SE, Strecker RE, Tate BA, Parker RA, Saper CB. Ventrolateral preoptic nucleus contains sleep-active, galaninergic neurons in multiple mammalian species. Neuroscience. 2002;115(1):285–94. Epub 2002/10/29. . 12401341 10.1016/s0306-4522(02)00308-1

[8] Braun AR, Balkin TJ, Wesenten NJ, Carson RE, Varga M, Baldwin P, et al Regional cerebral blood flow throughout the sleep-wake cycle. An H2(15)O PET study. Brain. 1997;120 (Pt 7):1173–97. Epub 1997/07/01. . 9236630 10.1093/brain/120.7.1173

[9] Maquet P. Functional neuroimaging of normal human sleep by positron emission tomography. J Sleep Res. 2000;9(3):207–31. Epub 2000/09/30. . 11012860 10.1046/j.1365-2869.2000.00214.x

[10] Fiset P, Paus T, Daloze T, Plourde G, Meuret P, Bonhomme V, et al Brain mechanisms of propofol-induced loss of consciousness in humans: a positron emission tomographic study. J Neurosci. 1999;19(13):5506–13. Epub 1999/06/23. . 10377359 10.1523/JNEUROSCI.19-13-05506.1999 PMC6782309

[11] Bonhomme V, Fiset P, Meuret P, Backman S, Plourde G, Paus T, et al Propofol anesthesia and cerebral blood flow changes elicited by vibrotactile stimulation: a positron emission tomography study. J Neurophysiol. 2001;85(3):1299–308. Epub 2001/03/15. doi: 10.1152/jn.2001.85.3.1299 . 11247998 10.1152/jn.2001.85.3.1299

[12] Kaisti KK, Langsjo JW, Aalto S, Oikonen V, Sipila H, Teras M, et al Effects of sevoflurane, propofol, and adjunct nitrous oxide on regional cerebral blood flow, oxygen consumption, and blood volume in humans. Anesthesiology. 2003;99(3):603–13. Epub 2003/09/10. . 12960544 10.1097/00000542-200309000-00015

[13] Pal D, Silverstein BH, Lee H, Mashour GA. Neural Correlates of Wakefulness, Sleep, and General Anesthesia: An Experimental Study in Rat. Anesthesiology. 2016;125(5):929–42. Epub 2016/10/19. doi: 10.1097/ALN.0000000000001342 ; PubMed Central PMCID: PMCPMC5069172. 27617688 10.1097/ALN.0000000000001342 PMC5069172

[14] Mhuircheartaigh RN, Rosenorn-Lanng D, Wise R, Jbabdi S, Rogers R, Tracey I. Cortical and subcortical connectivity changes during decreasing levels of consciousness in humans: a functional magnetic resonance imaging study using propofol. Journal of Neuroscience. 2010;(1529-2401 (Electronic)). Epub 2010. doi: 10.1523/JNEUROSCI.5516-09.2010 20610743 10.1523/JNEUROSCI.5516-09.2010 PMC6632477

[15] Gili T, Saxena N, Diukova A, Murphy K, Hall JE, Wise RG. The thalamus and brainstem act as key hubs in alterations of human brain network connectivity induced by mild propofol sedation. J Neurosci. 2013;33(9):4024–31. Epub 2013/03/01. doi: 10.1523/JNEUROSCI.3480-12.2013 . PubMed Central PMCID: PMCPmc4162411. 23447611 10.1523/JNEUROSCI.3480-12.2013 PMC4162411

[16] Picchioni D, Pixa ML, Fukunaga M, Carr WS, Horovitz SG, Braun AR, et al Decreased connectivity between the thalamus and the neocortex during human nonrapid eye movement sleep. Sleep. 2014;37(2):387–97. Epub 2014/02/06. doi: 10.5665/sleep.3422 ; PubMed Central PMCID: PMCPMC3900615. 24497667 10.5665/sleep.3422 PMC3900615

[17] Gili T, Saxena N, Diukova A, Murphy K, Hall JE, Wise RG. The Thalamus and Brainstem Act As Key Hubs in Alterations of Human Brain Network Connectivity Induced by Mild Propofol Sedation. The Journal of Neuroscience. 2013;33(9):4024–31. doi: 10.1523/JNEUROSCI.3480-12.2013 23447611 10.1523/JNEUROSCI.3480-12.2013 PMC4162411

[18] Adapa RM, Axell RG, Mangat JS, Carpenter TA, Absalom AR. Safety and performance of TCI pumps in a magnetic resonance imaging environment. Anaesthesia. 2012;67(1):33–9. Epub 2011/10/07. doi: 10.1111/j.1365-2044.2011.06917.x . 21972913 10.1111/j.1365-2044.2011.06917.x

[19] Ramsay MA, Savege TM, Simpson BR, Goodwin R. Controlled sedation with alphaxalone-alphadolone. Br Med J. 1974;2(5920):656–9. Epub 1974/06/22. ; PubMed Central PMCID: PMCPmc1613102. 4835444 10.1136/bmj.2.5920.656 PMC1613102

[20] Allen PJ, Josephs O, Turner R. A method for removing imaging artifact from continuous EEG recorded during functional MRI. Neuroimage. 2000;12(2):230–9. Epub 2000/07/29. doi: 10.1006/nimg.2000.0599 . 10913328 10.1006/nimg.2000.0599

[21] Novelli L, Ferri R, Bruni O. Sleep classification according to AASM and Rechtschaffen and Kales: effects on sleep scoring parameters of children and adolescents. J Sleep Res. 2010;19(1 Pt 2):238–47. Epub 2009/11/17. doi: 10.1111/j.1365-2869.2009.00785.x . 19912509 10.1111/j.1365-2869.2009.00785.x

[22] Zang Y, Jiang T, Lu Y, He Y, Tian L. Regional homogeneity approach to fMRI data analysis. Neuroimage. 2004;22(1):394–400. Epub 2004/04/28. doi: 10.1016/j.neuroimage.2003.12.030 . 15110032 10.1016/j.neuroimage.2003.12.030

[23] Margulies DS, Vincent JL, Kelly C, Lohmann G, Uddin LQ, Biswal BB, et al Precuneus shares intrinsic functional architecture in humans and monkeys. Proc Natl Acad Sci U S A. 2009;106(47):20069–74. Epub 2009/11/12. doi: 10.1073/pnas.0905314106 ; PubMed Central PMCID: PMCPmc2775700. 19903877 10.1073/pnas.0905314106 PMC2775700

[24] Cavanna AE, Trimble MR. The precuneus: a review of its functional anatomy and behavioural correlates. Brain. 2006;129(Pt 3):564–83. Epub 2006/01/10. doi: 10.1093/brain/awl004 . 16399806 10.1093/brain/awl004

[25] Mulert C, Menzinger E, Leicht G, Pogarell O, Hegerl U. Evidence for a close relationship between conscious effort and anterior cingulate cortex activity. International Journal of Psychophysiology. 2005;56(1):65–80. doi: 10.1016/j.ijpsycho.2004.10.002 15725491 10.1016/j.ijpsycho.2004.10.002

[26] Upadhyay J, Baker SJ, Chandran P, Miller L, Lee Y, Marek GJ, et al Default-mode-like network activation in awake rodents. PLoS One. 2011;6(11):e27839 Epub 2011/11/30. doi: 10.1371/journal.pone.0027839 ; PubMed Central PMCID: PMCPmc3220684. 22125628 10.1371/journal.pone.0027839 PMC3220684

[27] Liu X, Pillay S, Li R, Vizuete JA, Pechman KR, Schmainda KM, et al Multiphasic modification of intrinsic functional connectivity of the rat brain during increasing levels of propofol. Neuroimage. 2013;83:581–92. Epub 2013/07/16. doi: 10.1016/j.neuroimage.2013.07.003 ; PubMed Central PMCID: PMCPmc3815996. 23851326 10.1016/j.neuroimage.2013.07.003 PMC3815996

[28] Cavazzuti M, Porro CA, Barbieri A, Galetti A. Brain and spinal cord metabolic activity during propofol anaesthesia. Br J Anaesth. 1991;66(4):490–5. Epub 1991/04/01. . 2025477 10.1093/bja/66.4.490

[29] Yamada M, Nakao S, Sakamoto S, Takamori Y, Tamura Y, Mochizuki-Oda N, et al Propofol acts at the sigma-1 receptor and inhibits pentazocine-induced c-Fos expression in the mouse posterior cingulate and retrosplenial cortices. Acta Anaesthesiol Scand. 2006;50(7):875–81. Epub 2006/08/02. doi: 10.1111/j.1399-6576.2006.01033.x . 16879472 10.1111/j.1399-6576.2006.01033.x

[30] Martuzzi R, Ramani R, Qiu M, Shen X, Papademetris X, Constable RT. A whole-brain voxel based measure of intrinsic connectivity contrast reveals local changes in tissue connectivity with anesthetic without a priori assumptions on thresholds or regions of interest. Neuroimage. 2011;58(4):1044–50. Epub 2011/07/19. doi: 10.1016/j.neuroimage.2011.06.075 ; PubMed Central PMCID: PMCPMC3183817. 21763437 10.1016/j.neuroimage.2011.06.075 PMC3183817

[31] Vargo J, Howard K, Petrillo J, Scott J, Revicki DA. Development and validation of the patient and clinician sedation satisfaction index for colonoscopy and upper endoscopy. Clin Gastroenterol Hepatol. 2009;7(2):156–62. Epub 2008/10/22. doi: 10.1016/j.cgh.2008.09.004 . 18930167 10.1016/j.cgh.2008.09.004

[32] Engbers FH, Sutcliffe N, Kenny G, Schraag S. Pharmacokinetic models for propofol: defining and illuminating the devil in the detail. Br J Anaesth. 2010;104(2):261–2; author reply 2–4. Epub 2010/01/21. doi: 10.1093/bja/aep385 . 20086066 10.1093/bja/aep385

[33] Chisholm CJ, Zurica J, Mironov D, Sciacca RR, Ornstein E, Heyer EJ. Comparison of electrophysiologic monitors with clinical assessment of level of sedation. Mayo Clin Proc. 2006;81(1):46–52. Epub 2006/01/28. doi: 10.4065/81.1.46 ; PubMed Central PMCID: PMCPmc1413967. 16438478 10.4065/81.1.46 PMC1413967

[34] Veselis RA, Feshchenko VA, Reinsel RA, Beattie B, Akhurst TJ. Propofol and thiopental do not interfere with regional cerebral blood flow response at sedative concentrations. Anesthesiology. 2005;102(1):26–34. Epub 2004/12/25. . 15618783 10.1097/00000542-200501000-00008

